# ESAT-6 and HspX Improve the Effectiveness of BCG to Induce Human Dendritic Cells-Dependent Th1 and NK Cells Activation

**DOI:** 10.1371/journal.pone.0075684

**Published:** 2013-10-09

**Authors:** Laura Marongiu, Marta Donini, Lara Toffali, Elena Zenaro, Stefano Dusi

**Affiliations:** Department of Pathology and Diagnostics, Section of General Pathology, University of Verona, Verona, Italy; Alabama State University, United States of America

## Abstract

The limited efficacy of the BCG vaccine against tuberculosis is partly due to the missing expression of immunogenic proteins. We analyzed whether the addition to BCG of ESAT-6 and HspX, two Mycobacterium tuberculosis (Mtb) antigens, could enhance its capacity to activate human dendritic cells (DCs). BCG showed a weak ability to induce DC maturation, cytokine release, and CD4^+^ lymphocytes and NK cells activation. The addition of ESAT-6 or HspX alone to BCG-stimulated DC did not improve these processes, whereas their simultaneous addition enhanced BCG-dependent DC maturation and cytokine release, as well as the ability of BCG-treated DCs to stimulate IFN-γ release and CD69 expression by CD4^+^ lymphocytes and NK cells. Addition of TLR2-blocking antibody decreased IL-12 release by BCG-stimulated DCs incubated with ESAT-6 and HspX, as well as IFN-γ secretion by CD4^+^ lymphocytes co-cultured with these cells. Moreover, HspX and ESAT-6 improved the capacity of BCG-treated DCs to induce the expression of memory phenotype marker CD45RO in naïve CD4^+^ T cells. Our results indicate that ESAT-6 and HspX cooperation enables BCG-treated human DCs to induce T lymphocyte and NK cell-mediated immune responses through TLR2-dependent IL-12 secretion. Therefore ESAT-6 and HspX represent good candidates for improving the effectiveness of BCG vaccination.

## Introduction

Mycobacterium tuberculosis (Mtb), the etiological agent of tuberculosis, modulates dendritic cell (DC) and T lymphocyte functions in diverse ways. Treatment of immature monocyte-derived DCs with Mtb elicits the formation of mature DCs, which produce several cytokines and activate T lymphocytes [1]. However, Mtb also alters DC differentiation [2], maturation and cytokine secretion [3]–[4], in order to survive inside the host organism. Mtb secretes numerous proteins that subvert host defenses [5] and impair the development of protective immunity [6]–[7]. Among such are 16-kDa heat shock protein (HspX) (Rv2031c) [6] and early secreted antigenic target protein-6 (ESAT-6) (Rv3875) [7]. HspX, also known as α-crystallin, is secreted during the latency phase of mycobacterial growth and is required for the persistence of Mtb in the environment of the macrophage phagosome [8]. HspX also plays a role in slowing Mtb growth [9] and generates IFN-γ-producing T cells in the peripheral blood mononuclear cells (PBMC) of Mtb-exposed individuals [8].

ESAT-6, a highly immunogenic secreted protein [10], is able to lyse alveolar epithelial cells and macrophages [11], destabilize phagolysosomes [12], and activate the inflammasome [13]. Recently, Romagnoli et al. demonstrated that ESAT-6 is involved in the ability of Mtb to escape the human DC phagosome [14]. Also, ESAT-6 is known to induce the PBMC of tuberculosis-bearing patients to produce IFN-γ and chemokines [15]–[16]. Furthermore, recombinant DNA vaccine encoding ESAT-6 elicits a strong Th1 response in mice [17]. Vaccination with a fusion protein composed of ESAT-6 and Antigen 85B, a protein belonging to the Mtb Antigen 85 complex [18], activates DCs and Th1/Th17 cell responses in mouse models [19]–[21].

Collectively, these findings suggest that HspX and ESAT-6 may be promising candidates for vaccines against tuberculosis, although Wang et al. have found that high doses of ESAT-6 decrease Th1/Th17 cell activity [22], indicating that the optimal design of such vaccines requires further investigation to better characterize the effects these antigens have on immune cells.

Bacillus Calmette-Guerin (BCG), the only tuberculosis vaccine currently used, is a live, attenuated strain obtained from virulent Mycobacterium bovis, closely related to Mtb. Its attenuation results in the deletion of region of difference 1 (RD1), a 9.5 Kb region encoding nine genes, including ESAT-6. RD1 is absent from all BCG substrains but present in virulent M. bovis and M. tuberculosis [7]. Given that BCG often fails to protect against pulmonary tuberculosis in adults [23], recent research has been focused on improving the effectiveness of BCG. One way to do this is by introducing Mtb antigens absent from BCG, such as ESAT-6. Another is to induce the overexpression of immunogenic proteins not expressed throughout all phases of Mtb infection, such as HspX [24]. Majlessi et al. demonstrated that the reintroduction of RD1 into BCG improved its capacity to protect mice against Mtb challenge [25]. Similarly, HspX augments the immune stimulatory effect of BCG. In fact, HspX based vaccines enhance the ability of BCG to stimulate immune response in mice, providing a better protection against Mtb [8], [26]–[28].

Therefore, many reports indicate that ESAT-6 and HspX improve the capacity of BCG to activate the host immune system against Mtb in mouse models. However, little is known about the effect of these antigens on human immune cells stimulated with BCG. Indeed, further studies are needed to elucidate a possible cooperation between ESAT-6, HspX, and BCG in human DCs function and to investigate the dual role of Mtb antigens as vaccine candidates and as virulence factors inhibiting the immune system [29]. For these reasons, we analyzed the effects of BCG, ESAT-6 and HspX on the maturation and function of human primary DCs. In particular, we investigated whether the addition of ESAT-6 and HspX antigens, alone or in combination, could improve the ability of BCG to induce DC maturation and/or cytokine release, as well as the capacity of BCG- and antigen-treated DCs to stimulate T lymphocytes and Natural Killer (NK) cells. Also we sought to identify the DC receptors and cytokines responsible for the effects of ESAT-6 and HspX. Our results indicate that ESAT-6 and HspX represent possible candidates for improving the effectiveness of BCG on human immune cells.

## Materials and Methods

### Reagents and Antibodies

RPMI 1640 and low-endotoxin fetal bovine serum (FBS) were obtained from Lonza (Walkersville, MD). Recombinant human granulocyte-macrophage colony-stimulating factor (GM-CSF) and human IL-4 were purchased from Miltenyi Biotec (Bergisch Gladbach, Germany). Gamma-irradiated whole cells of M. tuberculosis (strain H37Rv) were obtained through BEI Resources, NIAID, (NIH NR-14819); α-crystallin (Gene Rv2031c), a purified native protein from M. tuberculosis (strain H37Rv, NR-14860) was provided by NIH Biodefense and Emerging Infections Research Resources Repository, NIAID, NIH; M. *bovis* BCG kindly provided by Dr. G. Batoni (Dept. Of Experimental Pathology, Medical Biotechnology, Infectivology and Epidemiology, University of Pisa, Italy) was prepared as described in [30] and killed at 55°C for 30 minutes; rdESAT-6 was provided by Statens Serum Institut (Copenhagen, Denmark). Ultrapure lipolysaccharide (LPS) from E. coli (0111: B4 strain) and palmitoyl-3-cysteine-serine-lysine-4 (Pam3CSK4) were purchased from InvivoGen (San Diego, CA). All of the above reagents contained less than 0.125 endotoxin units/ml, as measured by the Limulus amebocyte lysate assay (Microbiological Associates, Walkersville, MD). Flow cytometric analysis was performed using the following mouse anti-human antibodies: CD83 (HB15e), CD4 (SK3) and CD1a (HI149) (Becton Dickinson, San Jose, CA); CD56 (HCD56), CD69 (FN50), CD86 (T2.2), HLA-DR (L243) and IFN-γ (4S.B3) (Biolegend, San Diego, CA); CD4 (VIT4), CD45RO (UCHLI), CD45RA (T6D11) (Miltenyi Biotec); IL-17AF (20LJS09) (eBioscience, San Diego, CA). The blocking antibodies were: anti- Toll-like Receptor (TLR) 2 (T2.5) (eBioscience, San Diego, CA); anti-IL12p70 (20C2) [31]–[32] kindly provided by Dr. G. Trinchieri (Center for Cancer Research, NCI, Frederick, MD). Monoclonal mouse IgG1 (eBioscence) was used as the isotype control antibody.

### Preparation and Culture of DCs, Lymphocytes and NK Cells

After written informed consent and upon approval of the ethical committee, human blood was collected from healthy volunteers at the blood bank of the University of Verona. Monocytes were isolated from buffy coats by Ficoll-Hypaque and Percoll (GE Healthcare Life Science) density gradients and purified using the human monocyte isolation kit II (Miltenyi Biotec), as previously described [1]. The final monocyte population was 99% pure, as measured by FACS analysis. To generate immature DCs, monocytes were incubated at 37°C in 5% CO2 for 5–6 days at 1×106/ml in 6-well tissue culture plates (Greiner, Nürtingen, Germany) in RPMI 1640, supplemented with heat-inactivated 10% low endotoxin FBS, 2 mM L-glutamine, 50 ng/ml GM-CSF, and 20 ng/ml IL-4. The final DC population was 98% CD1a^+^, as measured by FACS analysis.

NK cells and autologous total and naïve CD4^+^ T cells were isolated from the lymphocyte fraction of the Percoll gradient with EasySep™ Negative Selection Human Cell Enrichment kits (StemCell Technologies, Vancouver, Canada). The final populations were 98% pure, as measured by FACS analysis. To preserve T cells during differentiation of monocytes into DCs, the cells were spinned down, resuspended in freezing medium (low endotoxin FBS +10% DMSO), and kept in a liquid nitrogen freezer.

To induce cell maturation and cytokine release, DCs were treated for 24 hrs with: Mtb (50 µg/ml), Pam3CSK4 (10 µg/ml), LPS (100 ng/ml), BCG (50 µg/ml) alone or combined with HspX (10 µg/ml) and/or ESAT-6 (10 µg/ml). For experiments with blocking antibodies, immature DCs were pre-incubated for 15 min at room temperature with anti-IL-12p70 or anti-TLR2 antibodies and with an isotype antibody IgG1. In order to study their effects on T lymphocytes, mature DCs were co-cultured for 7 days with total CD4^+^ T cells or for 9 days with naïve CD4^+^ T cells. The DCs:T-cell ratio was 1∶10. NK cells were incubated for 24 hrs with conditioned media (added to 1∶1 ratio) from DCs treated with Mtb (50 µg/ml) or BCG (50 µg/ml) alone or combined with HspX (10 µg/ml) and/or ESAT-6 (10 µg/ml). For the experiments with blocking antibodies, the supernatants were pre-incubated for 15 minutes at room temperature with anti-IL-12p70 and with an isotype antibody IgG1.

### ELISA Assay

Cytokine production in culture supernatants was determined by ELISA according to the manufacturer’s instructions: IL-6 (range 8–800 pg/ml) purchased from ImmunoTools GmbH, (Friesoythe, Germany); IL-12 (range 4–500 pg/ml), IL-1β (range 4–500 pg/ml), IL-23 (range 15–2000 pg/ml), TNF-α (range 4–500 pg/ml), IL-10 (range 2–300 pg/ml), IFN-γ (range 4–500 pg/ml), IL-17AF (range 30–4000 pg/ml), purchased from eBioscience (San Diego, CA).

### Flow Cytometric Analysis

For surface staining, cells were washed twice with PBS salt solution and incubated for 30 min with 10% human serum to prevent non-specific binding. For direct immunofluorescence staining, mouse anti-human CD1a, CD83, CD86, HLA-DR, CD69, CD56, CD4, CD45RO, and CD45RA were used (see reagents). Following stimulation with 20 ng/ml PMA, 1 µM ionomycin and 10 µg/ml brefeldin A (Biolegend) for the final 6 hrs of culture [33], cytokine intracellular staining of T cells was performed. After staining with a fluorescent-conjugated antibody anti-CD4, the cells were incubated with fixation/permeabilization buffer (420801 and 421002, Biolegend). Subsequently, they were stained with anti-IL17AF and IFN-γ fluorescent-conjugated antibodies (see reagents). Annexin-V (Roche Applied Science, Indianapolis, IN) was used to detect apoptotic cells. Samples were acquired on a seven-color MACSQuant Analyzer (Miltenyi Biotec) and analyzed with FlowJo 10 (TreeStar).

### Statistical Analysis

Data are expressed as the mean^+^SD. Statistical analyses, including Student’s t test and one-way ANOVA with Bonferroni test, were performed using SigmaStat 3.0 for Windows (Systat Software, San Jose, CA).

## Results

### ESAT-6 and HspX Improve the Ability of BCG to Stimulate Human DC Maturation and Pro-inflammatory Cytokine Release

**Figure 1:**
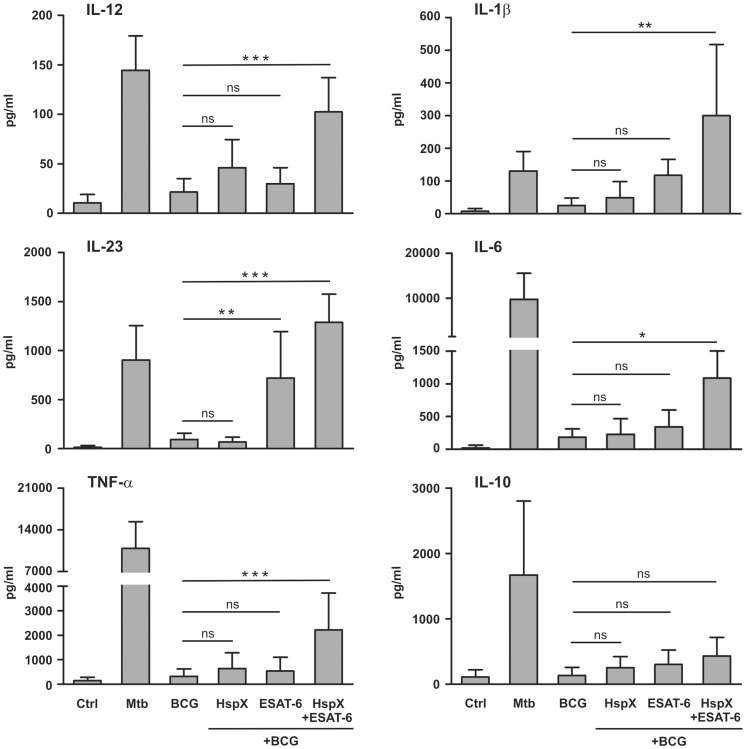
Effect of HspX and ESAT-6 on BCG-induced cytokine secretion by DCs. Monocytes were treated (5 days) with 50 ng/ml GM-CSF and 20 ng/ml IL-4 to obtain immature DCs, that were subsequently cultured (24 hrs) in the absence (CTRL) or presence of 50 µg/ml BCG, alone or combined with 10 µg/ml HspX and/or 10 µg/ml ESAT-6. DCs were also cultured with 50 µg/ml Mtb as a positive control. Release of the indicated cytokines in culture supernatants was evaluated by ELISA. Results are expressed as the mean value+SD of seven independent experiments. Statistical analysis: DCs treated with BCG alone vs BCG plus HspX and ESAT-6 added alone or in combination; ns P>0.05, *P<0.05, **P<0.01, ***P<0.001.

The interaction of DCs with pathogenic microorganisms or their derivatives elicits the production of various cytokines that orchestrate the immune response [34]. We examined the capacity of BCG, ESAT-6 and HspX to induce cytokine secretion by DCs. For this purpose, monocytes were cultured for 5 days with GM-CSF and IL-4 to obtain immature DCs. The latter cells were challenged with BCG, ESAT-6 or HspX, as well as with Mtb as a positive control [1]. The bacteria and antigen doses were selected on the basis of preliminary dose-response experiments (results not shown). After 24-hr treatment, culture supernatants were collected and cytokine secretion was analyzed by ELISA. We found that BCG induced a weak release of these cytokines (Fig. 1). DCs challenge with a purified HspX and/or a recombinant ESAT-6 protein did not affect the cytokine production (results not shown). As reintroduction of the ESAT-6 gene restores the ability of BCG to activate mouse immune cells [25], we wanted to determine whether the addition of the ESAT-6 protein, alone or simultaneously with HspX, which also augments the immune stimulatory effects of BCG in mice [8], [24], could increase human DC response to BCG stimulation. We found that the addition of HspX alone to BCG-treated DCs did not significantly influence cytokine release, whereas ESAT-6 increased the secretion of IL-23 but not the other cytokines (Fig. 1). Interestingly, the simultaneous addition of HspX and ESAT-6 to BCG-stimulated DCs significantly increased the secretion of IL-12, IL-1β, IL-23, IL-6, and TNFα but not IL-10, as compared to DCs challenged with BCG alone or when combined with either antigen separately (Fig. 1).

**Figure 2:**
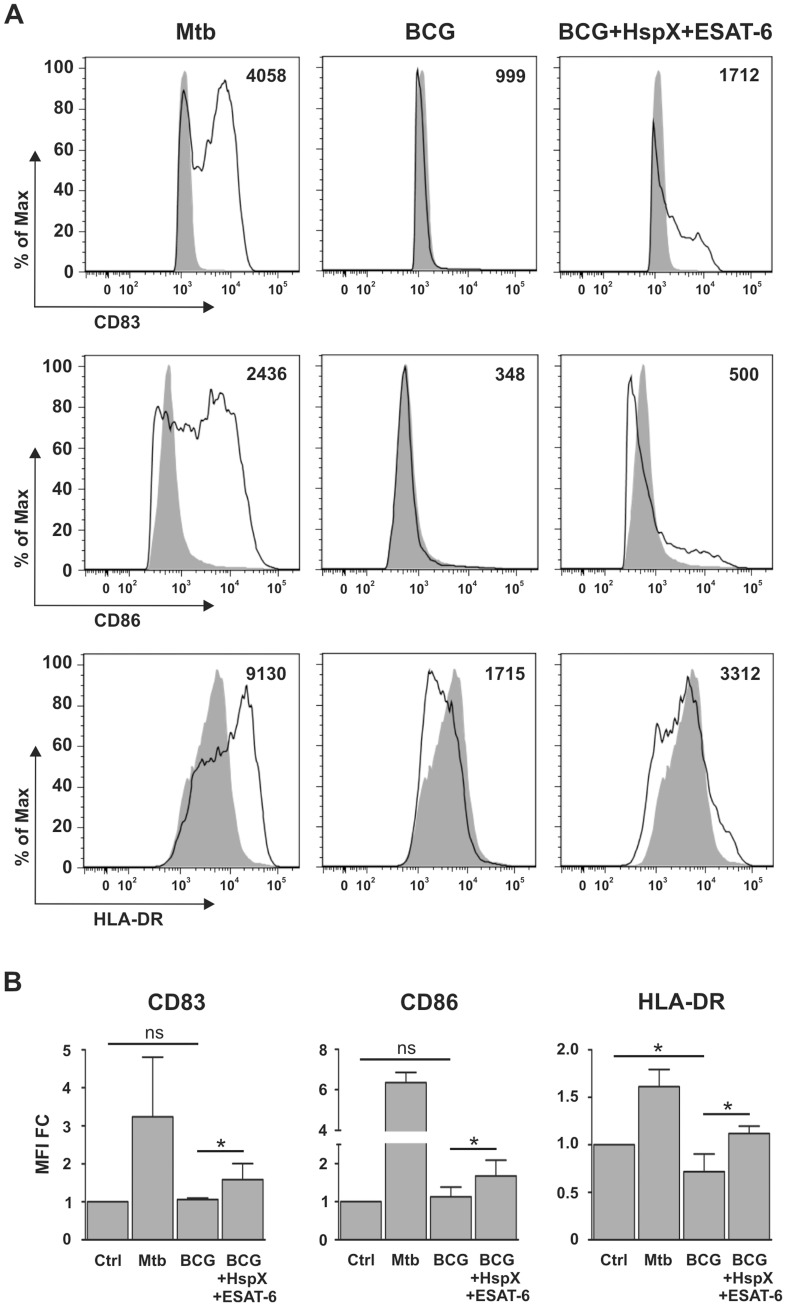
Effect of HspX and ESAT-6 on BCG-elicited DC maturation. DCs were treated with Mtb as a positive control and with BCG alone or combined with HspX and ESAT6. Cells were collected after 24(A) Histograms illustrate CD83, CD86 and HLA-DR surface expression in CD1a^+^ cells and the MFI. Filled histograms represent the control, open histograms indicate treated cells. One of four different experiments is presented. (B) Bar graphs show the CD83, CD86 and HLA-DR MFI value of the four experiments expressed as fold change (MFI FC) over control (CTRL). Statistical analysis: CTRL vs BCG alone; BCG alone vs BCG plus HspX and ESAT6; ns P>0.05, *P<0.05.

The interaction between immature DCs and pathogens induces their maturation, enabling DCs to activate immune effectors cells [34]. This leads to the formation of DCs with increased CD83, CD86 and HLA-DR expression on the membrane surface [35]–[36]. Consequently, we analyzed the maturation of the DCs already used for ELISA (Fig. 1) and found that BCG did not induce significant CD83 and CD86 up-regulation and that it inhibited HLA-DR basal expression, as compared to untreated DCs (Fig. 2A and B). However, the simultaneous addition of ESAT-6 and HspX to BCG-treated DCs significantly increased CD83, CD86, and HLA-DR expression, as compared to DCs incubated with BCG alone (Fig. 2A and B). DCs did not mature upon cell stimulation with ESAT-6 or HspX in the absence of BCG nor did they mature following cell stimulation with BCG and either ESAT-6 or HspX alone (results not shown).

### ESAT-6 and HspX Enable BCG-treated DCs to Activate CD4^+^ Lymphocytes

**Figure 3:**
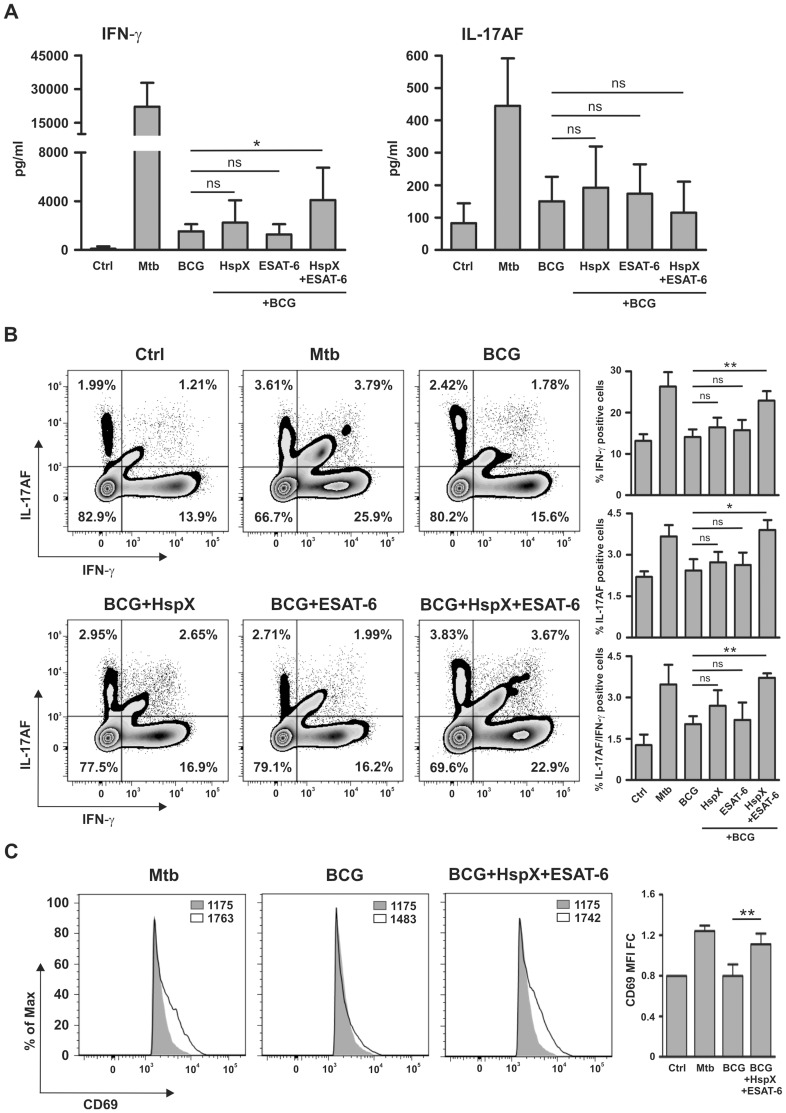
ESAT-6 and HspX enable BCG-stimulated DCs to elicit IFN-γ secretion and to enhance CD69 expression in CD4^+^ T lymphocytes. DCs were stimulated for 24-6 and/or HspX, and then co-cultured with autologous CD4^+^ T lymphocytes for 7 days. (A) Evaluation of IFN-γ and IL-17AF secretion in culture supernatants by ELISA. Results are expressed as the mean+SD of seven independent experiments. Statistical analysis: DCs treated with BCG alone vs BCG plus HspX and/or ESAT-6; ns P>0.05, *P<0.05. (B) Evaluation of IFN-γ and IL-17AF-production detected by intracellular staining and analyzed by FACS in Annexin V^−/^CD4^+^/CD1a^−^ cells. Zebra plots illustrate one representative experiment and the percentage of positive cells (left); bar graphs show the mean+SD of three independent experiments (right). Statistical analysis: DCs treated with BCG alone vs BCG plus HspX and/or ESAT-6; ns P>0.05, *P<0.05, **P<0.01. (C) Flow cytometric analysis of CD69 expression in Annexin V^−/^CD4^+^/CD1a^−^ cells. Panels illustrating one representative experiment with the MFI are shown on the left. Filled histograms represent the control, open histograms represent treated cells. Bar graphs representing the MFI mean value+SD of three experiments expressed as fold change over control (MFI FC) are shown on the right. Statistical analysis: DCs treated with BCG vs BCG/HspX/ESAT-6; **P<0.01.

Mature DC are known to regulate the activity of T lymphocytes which play a prominent role in defensive mechanisms against tuberculosis [37]. As ESAT-6 and HspX improved the BCG-dependent DC cytokine release and maturation (Fig. 1 and 2), we hypothesized that these antigens could influence the capacity of DCs to regulate T lymphocyte activity. Therefore, we investigated whether treatment with BCG, HspX and/or ESAT-6 would enable DCs to induce Th1 and/or Th17 response. For this purpose, DCs induced to maturation with Mtb or BCG, alone or combined with ESAT-6 and/or HspX, were co-cultured with autologous CD4^+^ lymphocytes. After 7 days the culture supernatants were assayed by ELISA for the presence of IFN-γ and IL-17AF. This time point was chosen on the basis of previous time course viability assays (results not shown). Figure 3A shows that DCs incubated with Mtb induced remarkable IFN-γ and IL-17AF production by T cells, whereas DCs stimulated with BCG, alone or in combination with either HspX or ESAT-6, showed a weak ability to activate these responses. However, DCs incubated with BCG and both HspX and ESAT-6 induced significantly higher IFN-γ secretion by CD4^+^ lymphocytes than that elicited by DCs challenged with BCG alone or when combined with either antigen separately (Fig. 3A). In contrast, IL-17AF secretion induced by DCs incubated with BCG/HspX/ESAT-6 was comparable to that observed when DCs were treated with BCG alone or in combination with either antigen separately.

Among the classical Th1 and Th17 cells responsible for IFN-γ and IL-17 production, respectively, a Th17/Th1 subset able to produce both IL-17 and IFN-γ has been discovered [38]. In order to identify the T cell subsets responsible for cytokine production (Fig. 3A), we performed IFN-γ and IL-17AF intracellular staining. Cell viability was evaluated by Annexin V staining. FACS analysis demonstrated that DC stimulated with BCG, alone or in combination with either HspX or ESAT-6 separately, showed a weak ability to induce Th1, Th17, and Th17/Th1 differentiation, whereas DC incubated with BCG/HspX/ESAT-6 induced a remarkable development of all these Th cell subsets. Notably, this effect was comparable to that obtained upon DCs challenge with Mtb (Fig. 3B). These results demonstrate that IFN-γ and IL-17AF, as detected by ELISA (Fig. 3A), are produced by Th1 and Th17, respectively, but also by Th17/Th1 cells. Although DCs incubated with BCG/HspX/ESAT-6 induced a significant increase in IL-17AF-producing cells as compared to BCG-treated DCs (Fig. 3B), the amount of IL-17AF detected in the culture media of these cells was not significantly different (Fig. 3A). This discrepancy could be due to the fact that a very low percentage of IL-17AF-producing cells is insufficient to generate significant IL-17AF protein secretion in culture media.

Control experiments with CD4^+^ lymphocytes stimulated with various combinations of BCG, ESAT-6 and HspX in the absence of DCs did not elicit IFN-γ or IL-17AF secretion (results not shown), suggesting that BCG, ESAT-6 and HspX do not directly modulate T cell activation. Moreover, DCs alone treated with various combinations of BCG, HspX, and ESAT-6 did not secrete IFN-γ or IL-17AF (results not shown), thus ruling out their contribution to cytokine production after co-culture of DCs with T lymphocytes.

Subsequently, we analyzed the effect of BCG, HspX and ESAT-6 on the ability of DCs to induce the expression of CD69, a well-known T lymphocyte activation marker, in CD4^+^ cells [39]. We found that BCG-treated DCs did not elicit CD69 expression, whereas the simultaneous addition of BCG, HspX and ESAT-6 up-regulated this activation marker in CD4^+^ cells co-cultured with DCs. Notably, this effect was comparable to that obtained upon DCs challenge with Mtb (Fig. 3C).

### TLR2-dependent IL-12 Secretion is Involved in CD4^+^ Lymphocyte Activation by DCs Stimulated with BCG, ESAT-6 and HspX

**Figure 4:**
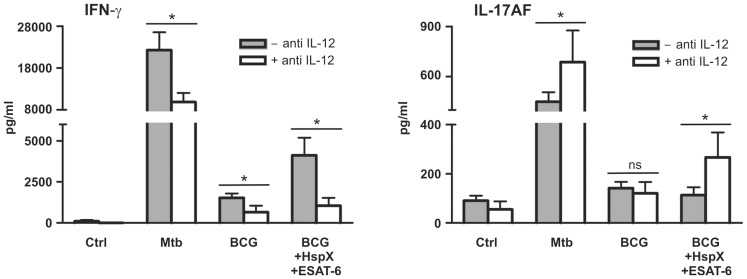
The ability of BCG, ESAT-6 and HspX-treated DCs to elicit IFN-γ secretion by CD4^+^ cell is mediated by IL-12. DCs pre-incubated (open bars) or not (filled bars) with 20 µg/ml IL-12p70 blocking antibody were stimulated for 24 hrs with Mtb or with BCG alone or with ESAT-6 and HspX and then co-cultured for 7 days with autologous CD4^+^ T lymphocytes. IFN-γ and IL-17AF secretion was analyzed by ELISA. Results are expressed as the mean+SD of five experiments. Statistical analysis: antibody-treated vs antibody-untreated cells; ns P>0.05, *P<0.05.

It is well accepted that IL-12 plays a key role in the induction of IFN-γ secretion by T lymphocytes [40]. We hypothesized that among the cytokines secreted upon DCs treatment with BCG/ESAT-6/HspX, IL-12 could be the one mainly involved in stimulating IFN-γ secretion by T lymphocytes (Fig. 3A). Consequently, we wanted to examine whether IL-12 blockage could affect IFN-γ secretion. To test this hypothesis, the cells were incubated with an antibody able to bind IL-12 p70 and, specifically, to block IL-12 without affecting IL-23 [31]–[32]. Figure 4 shows that IL-12 blockage decreased the ability of DCs challenged with BCG, alone or combined with HspX and ESAT-6, to induce IFN-γ secretion by CD4^+^ lymphocytes. Moreover, the antibody increased IL-17AF production by CD4^+^ lymphocytes cultured with DCs stimulated with BCG/HspX/ESAT-6 (Fig. 4). Similar results were obtained from control experiments with DCs incubated with the antibody and stimulated with Mtb (Fig. 4). An isotype matched antibody did not affect the capacity of DCs to modulate cytokine production by T cells (results not shown). These results indicate that ESAT-6 and HspX increase IFN-γ release by T lymphocytes mainly by enhancing IL-12 secretion by co-cultured BCG-conditioned DCs. Moreover, the ESAT-6- and HspX-dependent increase in IL-12 release inhibited IL-17AF secretion, shifting the lymphocytes toward a Th1 response characterized by a prevalent IFN-γ release.

**Figure 5:**
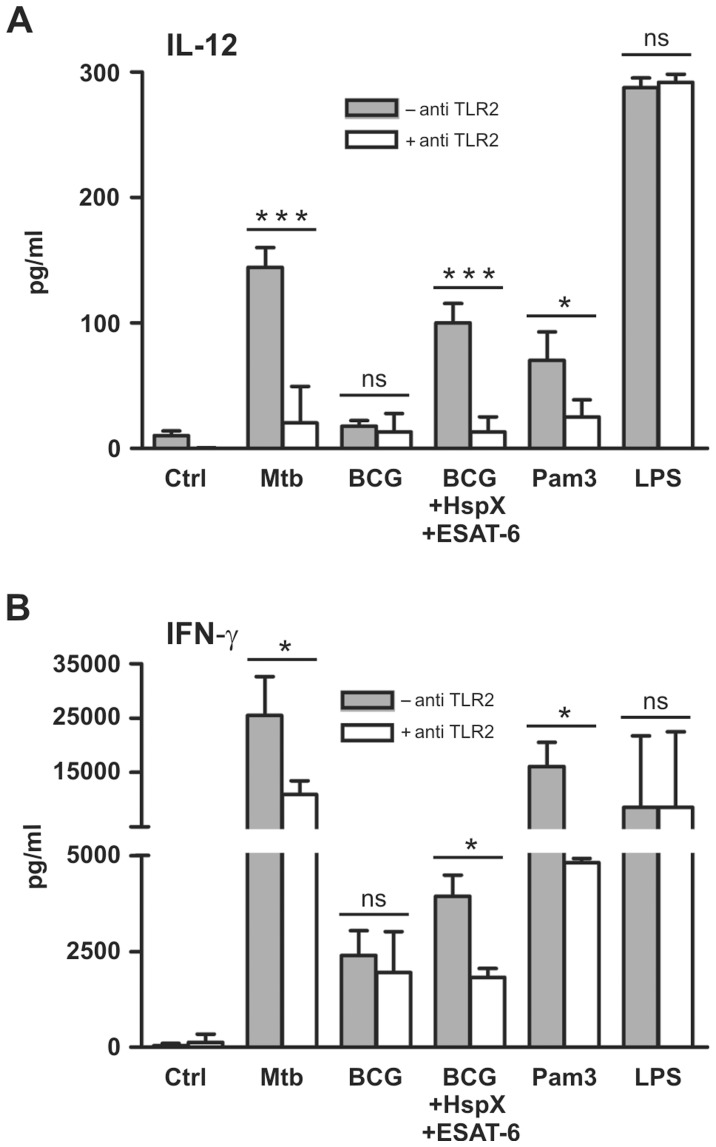
TLR2 is involved in IL-12-dependent IFN-γ secretion by CD4^+^ cells co-cultured with ESAT-6, HspX and BCG-treated DCs. DCs cultured in the absence (filled bars) or presence (open bars) of 5 µg/ml TLR2-blocking antibody were treated for 24 hrs with Mtb, BCG alone or combined with HspX and ESAT6, 10 µg/ml Pam3CSK4 (Pam3) or 100 ng/ml LPS. (A) Supernatants were collected and IL-12 release was analyzed by ELISA. Results are the mean value+SD of four experiments. Statistical analysis: antibody-treated vs antibody-untreated cells, ns P>0.05, *P<0.05, ***P<0.001. (B) DCs were co-cultured with autologous CD4^+^ T lymphocytes. After 7 days, culture supernatants were collected and analyzed by ELISA for IFN-γ release. Results are the mean+SD of three experiments. Statistical analysis: antibody-treated vs antibody-untreated cells, ns P>0.05, *P<0.05.

It has been reported that ESAT-6 [41]–[42] and some Mtb heat shock proteins [43] bind TLR2, which plays a critically important role in the interaction between DCs and mycobacteria [44]. Therefore, we explored whether a TLR2-blocking antibody could affect BCG, ESAT-6 and HspX cooperation. We found that the antibody reduced IL-12 release by BCG-treated DCs stimulated with ESAT-6 and HspX (Fig. 5A). The antibody also suppressed IL-12 release by both Mtb-treated DCs and control DCs stimulated with Pam3CSK4, a specific TLR2 agonist. In contrast, the antibody did not affect IL-12 production by DCs stimulated with LPS, a TLR4 agonist (Fig. 5A), indicating that it specifically blocks TLR2-dependent IL-12 release. An isotype matched antibody did not affect IL-12 release by DCs stimulated with BCG/ESAT-6/HspX, Mtb, Pam3CSK4 or LPS (results not shown). Interestingly, the TLR2-blocking antibody also decreased the ability of DCs incubated with Mtb, as well as with BCG/HspX/ESAT-6, to induce IFN-γ secretion by co-cultured CD4^+^ lymphocytes (Fig. 5B). Additionally, the capacity to induce IFN-γ secretion by CD4^+^ lymphocytes was inhibited by the antibody only in Pam3CSK4-stimulated DC and not in LPS-treated DCs (Fig. 5B).

### DCs Challenged with BCG, HspX and ESAT-6 Induce a Memory Phenotype in Naïve T Lymphocytes

**Figure 6:**
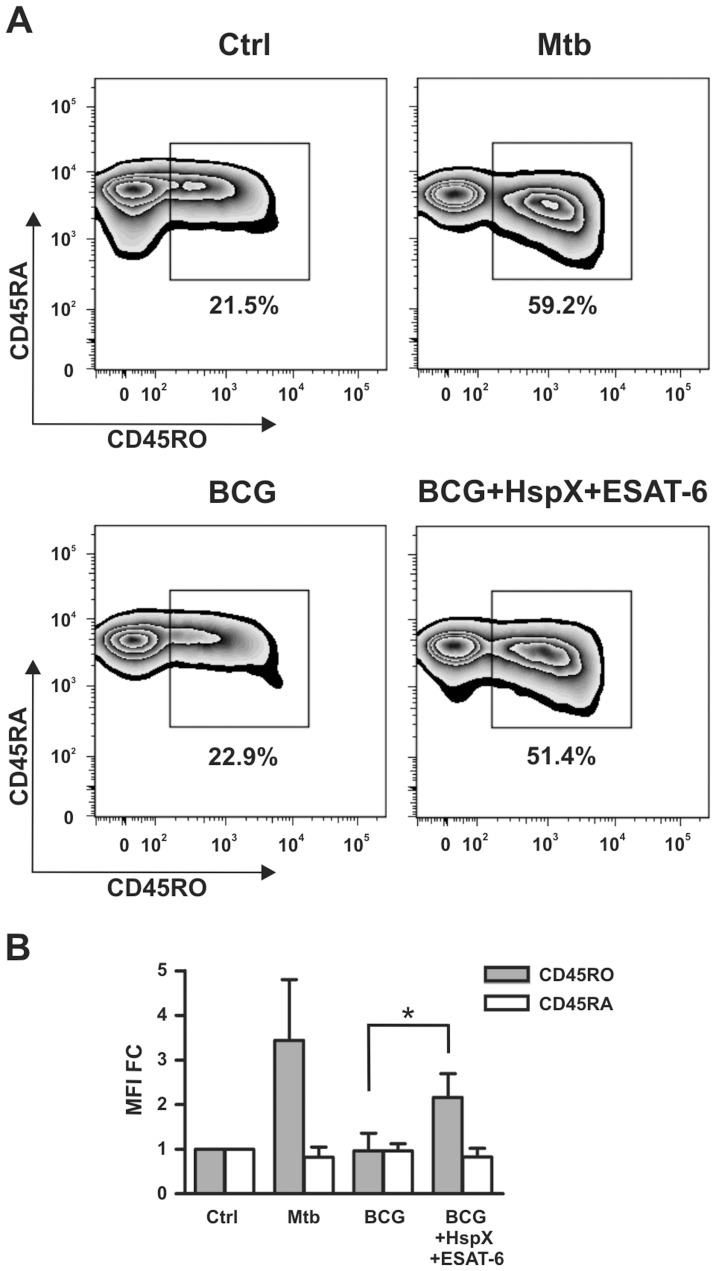
CD45RO and CD45RA modulation in CD4^+^ naïve T cells. DCs pre-treated for 24 hrs with Mtb or with BCG alone or combined with HspX and ESAT6 were co-cultured with purified CD4^+^ naïve T cells. After 9 days, the surface expression of CD45RO and CD45RA was analyzed by flow cytometry in Annexin V^−/^CD4^+^/CD1a^−^ cells. (A) Zebra plots illustrate one representative experiment with the percentage of CD45RO positive cells. (B) Bar graph shows the CD45RO and CD45RA MFI mean+SD of three experiments expressed as fold change over control (MFI FC). Statistical analysis: BCG vs BCG plus HspX and ESAT-6, *P<0.05.

Human CD4^+^ lymphocyte preparations contain both naïve and memory T cells. Hence, we examined whether DCs incubated with BCG/HspX/ESAT-6 were able to induce a memory phenotype in naïve T cells. For this purpose, we isolated naïve CD4^+^ T cells (CD45RA^+^/CD45RO^−^) and co-cultured them with DCs stimulated with BCG, alone or with HspX and ESAT-6, as well as with Mtb as a positive control. After 9 days, we analyzed by flow cytometry the expression of CD45RO and CD45RA, well-known memory and naïve T cell markers, respectively [45]. As shown in Figure 6A and B, BCG treatment did not lead to a significant expansion of CD45RO^+^ cells (22.9%), as compared to untreated cells (21.5%). Interestingly, we observed that the simultaneous addition of HspX and ESAT-6 enabled BCG-treated DCs to induce the expansion of memory CD4^+^ T cell population (51.4%) (Fig. 6A and B).

### ESAT-6 and HspX Improve the Ability of BCG-treated DCs to Activate NK Cells through Induction of IL-12 Release

**Figure 7:**
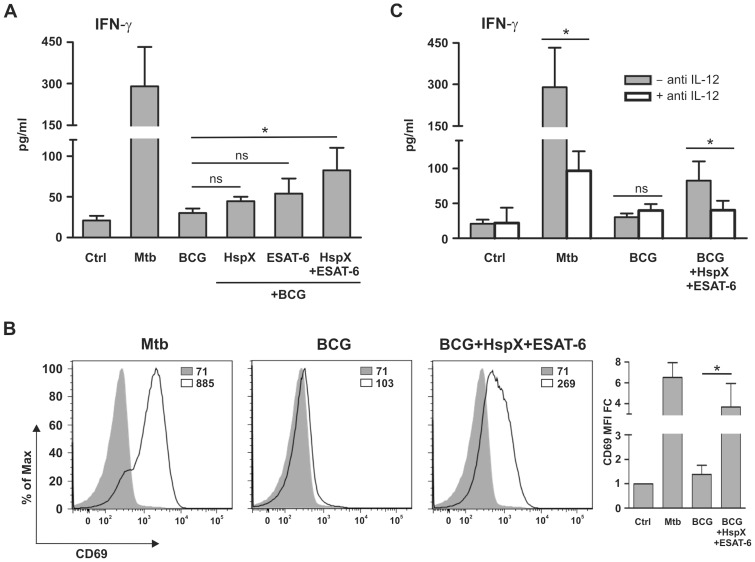
Effect of BCG plus ESAT-6 and HspX on DC-mediated IFN-γ release and CD69 expression by NK cells. Culture supernatants of DCs treated as described in Figure 1 were incubated without (filled bars) or with (open bars) 20 µg/ml IL-12-blocking antibody and then added to purified NK cells. After 24 hrs, IFN-γ release was measured by ELISA (A and C) and CD69 expression was analyzed by flow cytometry in CD56^+^ cells (B). (A) Results are the mean+SD of three experiments. Statistical analysis: NK cells stimulated with supernatants from BCG-treated DCs vs supernatants of BCG plus HspX and/or ESAT-6-treated DCs; ns P>0,05, *P<0.05. (B) Panels illustrate one representative experiment and the bar graph shows the MFI mean value+SD of three experiments expressed as fold change over control (MFI FC). Filled histograms represent the control; open histograms represent the treated cells. Statistical analysis: NK cells stimulated with supernatants from BCG-treated DCs vs supernatants of BCG/HspX/ESAT-6-treated DCs, *P<0.05; (C) Results are the mean+SD of three experiments. Statistical analysis: IL-12-blocking antibody-treated supernatants vs untreated supernatants; ns P>0,05, *P<0.05.

Soluble mediators released by mature DCs activate NK cells involved in the host defense against micobacteria [46]. We explored whether cytokines released in culture supernatants by DCs in the experimental conditions depicted in Figure 1 induced NK cell activation. For this purpose, NK cells were isolated and incubated with conditioned media collected from the cultures of DCs treated with BCG, alone or combined with ESAT-6 and/or HspX, as well as with Mtb as a positive control. IFN-γ secretion was analyzed by ELISA and expression of the CD69 activation marker was evaluated by FACS analysis. We found that the incubation with conditioned media from Mtb-treated DCs induced IFN-γ release by NK cells (Fig. 7A), whereas culture supernatants from DCs stimulated with BCG, added alone or in combination with either HspX or ESAT-6 separately, showed a slight ability to activate such a response (Fig. 7A). In contrast, media collected from cultures of DCs treated with BCG/HspX/ESAT-6 elicited a significantly higher IFN-γ release than that observed in the media from DCs incubated with BCG added alone or with the either antigen (Fig. 7A). The FACS analysis revealed that supernatants from DCs treated with Mtb or BCG/HspX/ESAT-6, but not with BCG alone, induced CD69 expression by NK cells (Fig. 7B). Direct NK cell stimulation with Mtb, BCG, ESAT-6 and HspX did not induce IFN-γ release or CD69 expression (results not shown), suggesting that NK cell activation is mediated by soluble agonists released by mycobacteria- and antigen-activated DCs.

As IL-12 plays an essential role in the NK cell activation [47], we analyzed the effect of the IL-12-blocking antibody, already used for the experiments shown in Fig. 4, on NK cell responses induced by culture media of DCs stimulated with BCG and the two antigens. Antibody addition to the media from DCs treated with Mtb or BCG/HspX/ESAT-6 decreased the ability of these supernatants to induce IFN-γ release by NK cells (Fig. 7C). An isotype matched antibody did not affect the capacity of culture media from DCs treated with Mtb or with BCG/HspX/ESAT-6 to stimulate IFN-γ release by NK cells (results not shown). These results indicate that ESAT-6 and HspX enhance NK cell activation by increasing IL-12 release from BCG-treated DCs.

## Discussion

Here we demonstrate that BCG shows a scarce ability to induce human DC maturation and cytokine release, which results in a subsequent weak capacity of DCs to induce CD4^+^ lymphocytes and NK cell activation. Our findings confirm previous results showing a weak immune cell response to BCG [29] which might, in part, explain why BCG vaccination does not produce strong and persistent protection against adult pulmonary tuberculosis. We have also shown that ESAT-6 and HspX, per se or when separately added to BCG-treated DC, do not significantly affect DC activity. However, ESAT-6 and HspX cooperate in increasing BCG-dependent DCs maturation and pro-inflammatory cytokine secretion, suggesting that the addition of HspX and ESAT-6 could attribute to BCG important immune stimulatory characteristics. Conversely, anti-inflammatory cytokine IL-10 secretion did not significantly increase, indicating that cooperation between ESAT-6 and HspX results in a preferential release of immune response-enhancing mediators. This indication is supported by the result that upon stimulation with both these antigens and BCG, DCs become capable to activate CD4^+^ lymphocytes and NK cells. Interestingly, this treatment rendered DCs able to induce a memory phenotype in naïve T lymphocytes, further corroborating the suggestion that HspX and ESAT-6 enhance the ability of BCG to activate immune responses.

Our findings are very important considering that the ability to elicit immunological memory is an essential requisite of vaccine components. Our data are consistent with previous reports that reintroduction of the ESAT-6 gene into BCG improves its capacity to protect mice against Mtb challenge [25].

It has been reported that the addition of either HspX [8] or ESAT-6 [15] alone activates IFN-γ production by human PBMC. These effects were obtained with cells from patients with tuberculosis, whereas healthy or BCG-vaccinated subjects were less or not responsive to HspX [8] or ESAT-6 [15]. It is conceivable, therefore, that in the absence of Mtb infection, stimulation with either HspX or ESAT-6 alone does not efficiently activate immune cells and/or boost BCG-induced cell responses. Our results suggest that a more effective stimulation might be obtained by treating human immune cells from healthy subjects with BCG and both the antigens.

It has been demonstrated that a recombinant DNA vaccine encoding ESAT-6 elicits a strong Th1 response in mouse models [17], and that HspX-based vaccines enhance the ability of BCG to stimulate immune response [8], [26]–[28]. In our study, however, neither ESAT-6 nor HspX alone activated immune cells on their own or when either was combined with BCG. This discrepancy indicates that, differently from murine cells, stimulation with both ESAT-6 and HspX is needed to induce human immune cell response.

Conversely, our findings are in line with previous results showing that vaccination with fusion protein composed of two Mtb antigens efficiently increases DCs and T cell response [19]–[21]. Importantly, we observed that DCs are necessary for the activation of T lymphocytes and NK cells by Mtb, BCG and antigens. This finding indicates that the effect of these agonists is mediated by DCs. A number of studies have suggested that DCs reinforce cellular immune response against Mtb. In fact, DCs are very well represented at the sites of Mtb infection, where they capture antigens, mature and migrate towards lymphoid organs in which they prime T cells through antigen presentation, cytokine secretion, and co-stimulatory molecule expression [48]. Our results corroborate these findings, highlighting the essential role of DCs in the mechanisms driving protective immunity against Mtb. We also found that conditioned media from DCs cultured with BCG and antigens activate NK cells, suggesting that soluble factors released by DCs are sufficient to activate NK cells. These results are crucial, given that NK cells are involved in the control of Mtb infection and activated by Mtb-treated DCs [46], [49]. We showed that among the many soluble factors secreted in culture media, IL-12 produced upon stimulation of BCG-treated DCs with HspX and ESAT-6 plays a central role in both CD4^+^ and NK cell stimulation. These findings agree with previous studies showing that IL-12 is the most important cytokine for T cell and NK cell activation [47].

Here we also report that inhibition of IL-12 release leads to increased IL-17AF secretion by T cells cultured with DCs stimulated with BCG, HspX and ESAT-6. Our results confirm previous findings that T cells shift from Th1 to Th17 production, depending on the type of cytokines present in the cell environment [38], [50]. In particular, IL-12 elicits IFN-γ secretion [51], whereas other cytokines, such as IL-1β, IL-23, and IL-6, promote and/or maintain both IL-17A and IL-17F release [52]–[54]. Our results show that DC treatment with BCG, HspX and ESAT-6 induces a remarkable increase in IL-1β, IL-23 and IL-6 secretion. In spite of this, the enhanced IL-12 release, obtained in the same experimental conditions, pushes T cells toward a Th1 response, characterized by IFN-γ secretion, and, at the same time, it inhibits Th17 response characterized by IL-17 production [55]. This finding is remarkable because Th1 cells are known to play an important role in host defense against Mtb [56]. Although the role of Th17 in host protection against tuberculosis has not been completely clarified, it has been shown that the IL-23/Th17 pathway is not crucial for the control of Mtb infection [57]; therefore, the shift from Th17 toward Th1 response, induced upon HspX and ESAT-6 treatment, might increase the effectiveness of immune response against Mtb.

Moreover, we have identified the receptors responsible for the effects of ESAT-6 and HspX on human DCs. Little is known about the receptors engaged by Mtb antigens. It has been demonstrated that ESAT-6 and some Mtb heat shock proteins bind TLR2 [41]–[43], which is involved in the interaction between DCs and mycobacteria [44]. Our study shows that TLR2 plays an important role in the mechanisms by which Mtb, ESAT-6 and HspX induce IL-12 release and subsequent Th1 response. The formality of TLR2 recruitment during the coordinated action of BCG and mycobacterial antigens remains to be characterized and will be the subject of future investigations. However, our results highlight that TLR2 participates in the biological events leading to the activation of immune defense against tuberculosis.

In conclusion, our findings demonstrate that HspX and ESAT-6 cooperate to enhance the capacity of human BCG-primed DCs to produce IL-12 which, in turn, induces an effective Th1 and NK cell response. Moreover, the cooperation of HspX, ESAT-6 and BCG in IL-12 production occurs through TLR2 receptor engagement. To our knowledge, this is the first evidence that HspX and ESAT-6 improve the ability of BCG to stimulate human DC-dependent activation of T lymphocytes and NK cells, suggesting that these antigens could be used to increase the immune system’s responsiveness to vaccination with BCG.
